# LSCC SNP variant regulates SOX2 modulation of VDAC3

**DOI:** 10.18632/oncotarget.24918

**Published:** 2018-04-27

**Authors:** Jacqueline Chyr, Dongmin Guo, Xiaobo Zhou

**Affiliations:** ^1^ Department of Cancer Biology, Wake Forest School of Medicine, Winston-Salem, NC 27157, USA; ^2^ Center for Bioinformatics and Systems Biology, Department of Radiology, Wake Forest School of Medicine, Winston-Salem, NC 27157, USA; ^3^ School of Biomedical Informatics, University of Texas Health Science Center at Houston, Houston, TX 77030, USA

**Keywords:** lung cancer, SOX2, eQTL, SNP, topologically associating domain

## Abstract

Lung squamous cell carcinoma (LSCC) is a genomically complex malignancy with no effective treatments. Recent studies have found a large number of DNA alterations such as SOX2 amplification in LSCC patients. As a stem cell transcription factor, SOX2 is important for the maintenance of pluripotent cells and may play a role in cancer. To study the downstream mechanisms of SOX2, we employed expression quantitative trait loci (eQTLs) technology to investigate how the presence of SOX2 affects the expression of target genes. We discovered unique eQTLs, such as rs798827-VDAC3 (FDR *p*-value = 0.0034), that are only found in SOX2-active patients but not in SOX2-inactive patients. SNP rs798827 is within strong linkage disequilibrium (*r*^2^ = 1) to rs58163073, where rs58163073 [T] allele increases the binding affinity of SOX2 and allele [TA] decreases it. In our analysis, SOX2 silencing downregulates VDAC3 in two LSCC cell lines. Chromatin conformation capturing data indicates that this SNP is located within the same Topologically Associating Domain (TAD) of VDAC3, further suggesting SOX2's role in the regulation of VDAC3 through the binding of rs58163073. By first subgrouping patients based on SOX2 activity, we made more relevant eQTL discoveries and our analysis can be applied to other diseases.

## INTRODUCTION

Lung cancer is the leading cause of cancer death in the United States with approximately 158,000 deaths in 2016 [1, 2]. Lung squamous cell carcinoma (LSCC) represents a large portion of lung cancers with over 60,000 new cases diagnosed each year [3, 4]. The five year survival rate for LSCC is only 12% [1, 4–6]. Unlike adenocarcinoma lung cancer (LUAD), the other major subtype of lung cancer, there are no targeted treatments for LSCC [7–9]. The higher molecular complexity of LSCC has hampered our understanding and the discovery of druggable targets for LSCC.

Recent studies, including the comprehensive analysis by The Cancer Genome Atlas (TCGA) network, have found a large number of DNA alterations [4]. The most notable alteration is amplification of chromosome 3q, which contains the SOX2 locus [10]. SRY (sex determining region Y)-box 2 (SOX2) is a stem cell transcription factor that plays a role in cell self-renewal and differentiation [11–14]. It has found to be amplified and/or overexpressed in 63% of LSCC at the transcript level and 80–90% at the protein level [10, 15, 16]. Other studies have shown that SOX2 overexpression is crucial in promoting LSCC formation *in vivo* upon loss of Pten and Cdkn2ab [17].

**Figure 1:**
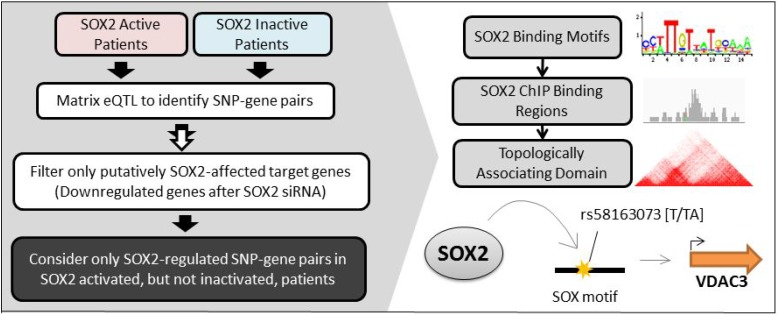
Brief overview of workflow Left: LSCC patients are first clustered into two groups: SOX2-active and SOX2-inactive based on their SOX2 gene expression, copy number variation, and methylation. Matrix eQTL analyses are performed on each group of patients to identify group-specific SNP-gene pairs. Only the eQTL pairs with genes that are downregulated after SOX2 silencing are included in our analysis. Only the SNP-gene pairs that are unique in SOX2 active patients are considered. Right: Further analyses on SOX2 motifs, ChIP-seq binding peaks, and topologically associating domains are conducted to validate a SOX2→ SNP→ Gene relationship.

There are different molecular mechanisms at play in SOX2-active LSCC patients compared to SOX2-inactive LSCC patients. SOX2 contains a DNA-binding region and regulates the expression of many downstream genes by binding to enhancer regions and facilitating in the remodeling of chromatin and subsequent initiation and transcription of target genes [18–20]. Other studies have found that single nucleotide polymorphisms (SNPs), located within the binding region of transcription factors, that can change the binding affinity of the factors and consequently the expression of their target genes [21–25]. Therefore, some SNPs, especially those located in SOX2 binding sequences, can alter SOX2's binding affinity to DNA. The increased or reduced binding of SOX2 to DNA can affect the transcription and expression of nearby target genes. Matrix eQTL allows us to identify local and distal eQTLs that are associated with the expression of target genes [26]. We can understand the downstream mechanism of SOX2 by identifying eQTLs in SOX2-active patients that are not in SOX2-inactive patients [26–28]. Analysis of flanking sequences of the eQTL or the SNPs in strong linkage equilibrium (LD) (*r*^2^ < 0.8) can elucidate which motifs the SNPs may alter. Additional analysis of ChIP-seq and high-throughput chromatin conformation capture (Hi-C) data can further confirm the interaction of SOX2 to specific regions of the genome and thereby its modulation of target genes [29, 30]. The general workflow is summarized in Figure 1. Our goal is to understand the complex mechanisms of LSCC in order to better develop targeted treatments that work for a subset of patients with SOX2 activation.

## RESULTS

### SOX2 in LSCC patients

**Figure 2:**
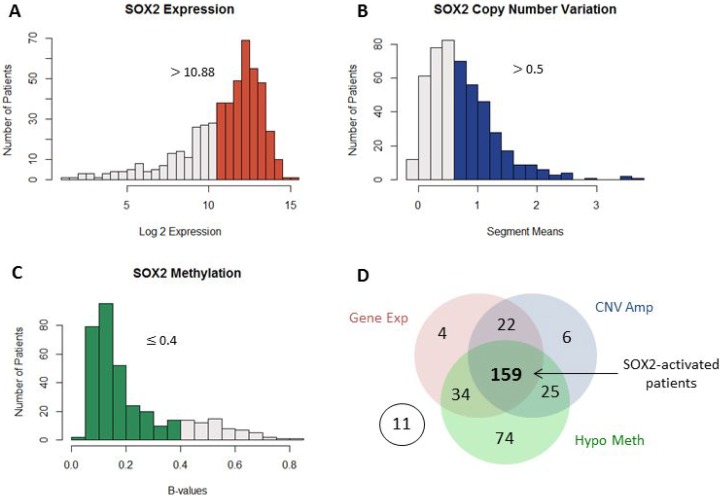
Patient genomic data Multiple genomic datatypes are used to group SOX2 patients into two groups. The highlighted bars in the histograms indicate patients with high SOX2 gene expression (**A**), SOX2 copy number amplification (**B**), and SOX2 promoter-region hypomethylation (**C**). Patients with SOX2 expression > 10.88, copy number segment means > 0.5, and methylation β-values ≤ 0.4 are considered as SOX2 active patients. A total number of 366 patents has all three datatypes available and were included in our analysis. (**D**) Of the 366 patients, 159 patients were considered as SOX2-active, all other patients are considered as SOX2-inactive.

Unlike LUAD, SOX2 is often seen amplified in LSCC patients [4]. Model-based analysis of regulation of gene expression (MARGE) confirms high regulatory potential of SOX2 in LSCC patients and suggests that it is a master regulator [31] (Supplementary Table 1). The increased presence of this transcription factor may lead to differential regulation of downstream genes. To understand SOX2's roles in LSCC, multiple types of data from TCGA was analyzed. Patients were first divided into two groups: SOX2-active and SOX2-inactive based on their gene expression, copy number variation, methylation of SOX2. For methylation, only the 14 SOX2 CpGs located within the promoter region of SOX2 are considered. These CpGs have high variance and are highly correlated with SOX2 gene expression (Supplementary Figure 1). Patients with activated SOX2 met all three of the following criteria (Figure 2A–2C):

1) SOX2 expression values greater than the mean (log2expression values > 10.87797)

2) SOX2 copy number variation is duplicated compared to normal (segment means > 0.5)

3) SOX2 promoter region is hypomethylated (methylation β-values ≤0.4)

Patients that did not meet all three criteria are considered as SOX2 inactive. A total of 366 LSCC patients had all three data types available and were included in our study. Out of the 366 patients, 219 had high SOX2 expression, 212 had SOX2 copy number amplification, and 292 patients had SOX2 hypomethylation. Although many patients met more than one criterion, only 159 patients which had all three criteria are grouped as SOX2-active and the other 196 patients are grouped as SOX2-inactive (Figure 2D).

### Matrix eQTL identifies SNP to VDAC3 expression association

**Figure 3:**
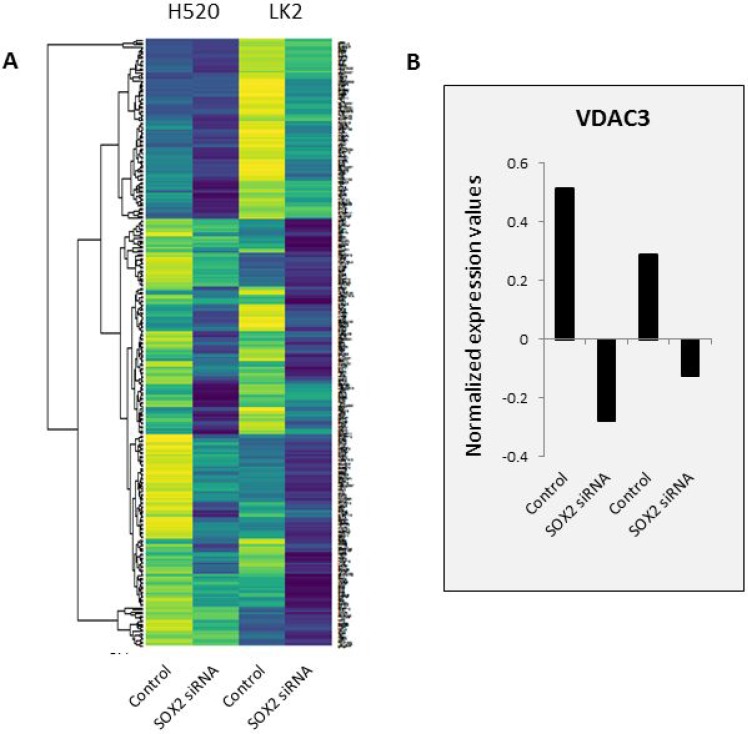
Gene expression profile of two SOX2-silenced LSCC cell lines SOX2 was knocked down in two LSCC cell lines: H520 and LK2 and gene expression was profiled using an array. (**A**) Top 266 downregulated genes are shown in a heat map. Downregulated genes are defined as genes with combined value differences between SOX2 siRNA and control > 1.0. Yellow color represents higher expression and dark blue color represents lower expression. (**B**) VDAC3's normalized expression values are shown as an example. All samples were normalized to 0.

Expression quantative trait loci (eQTL) analysis correlates SNP genotypes gene expression variations [26]. There are different sets of eQTLs in SOX2-active patients that are not in SOX2-inactive patients. Using a highly efficient and accurate eQTL analysis tool called Matrix eQTL [26, 28], we identified 686,251 cis SNP-gene pairs in the SOX2-active patients and 688,591 pairs in SOX2-inactive patients (*p* < 0.05) within 1 Mbps of each other. Since we are interested in the mechanism of SOX2, we focused on SNP-gene pairs that are putatively affected by SOX2 expression. Fang WT, *et al*. significantly knocked down SOX2 expression using siRNAs in two LSCC cell lines with high expression levels of SOX2 (LK2 and NCI-H20) [32]. The gene expression of the SOX2-knocked down cells was profiled using an array. After normalization, we conducted differential expression analysis and found 266 unique genes downregulated in SOX2 siRNA cells compared to control cells as shown in Figure 3A. Of the large number of SNP-gene pairs from our Matrix eQTL analysis, 8,546 SOX2-active and 8,956 SOX2-inactive pairs contained a gene that is downregulated upon silencing SOX2. Looking at the top results with FDR *p* < 0.01, only 16 and 38 cis SNP-gene pairs remain (Table 1 and Supplementary Table 2). For each pair, the expression of the gene is correlated with the genotype of the SNP. For example, patients with active SOX2, the genotypes for SNP rs798827 is significantly correlated with the expression of the gene VDAC3 (FDR *p*-value = 0.0034), where allele G is correlated with lower expression of VDAC3, and allele T is correlated with higher expression of VDAC3. Not only that, but VDAC3 is downregulated upon SOX2 silencing in two LSCC cell lines. Further analysis reveals one gene-pair overlap between SOX2 active and inactive cis-Matrix eQTL results. The correlation of this SNP-gene pair may be independent of SOX2 activity.

**Table 1:** eQTLs (FDR *p* value < 0.01) in SOX2-active patients

SNP	Allele A	Allele B	Gene	*P* value	FDR
rs4654947	C	T	NBPF3	4.60E-11	3.45E-07
rs9984519	C	T	IFNAR1	1.38E-07	5.59E-04
rs17420195	C	T	NBPF3	4.18E-07	1.45E-03
rs2465941	C	T	ZCCHC12	8.23E-07	2.60E-03
rs2290163	C	T	LMCD1	8.48E-07	2.67E-03
**rs798827**	**G**	**T**	**VDAC3**	**1.14E-06**	**3.43E-03**
rs4747471	A	G	MSRB2	1.22E-06	3.63E-03
rs16913776	C	G	RAB38	1.31E-06	3.83E-03
rs7624916	C	G	ARL6IP5	1.48E-06	4.27E-03
rs10968209	A	G	MOBKL2B	1.49E-06	4.27E-03
rs16850158	A	G	ALCAM	1.87E-06	5.14E-03
rs12057041	C	T	MOBKL2B	3.20E-06	7.98E-03
rs10968456	C	T	MOBKL2B	3.20E-06	7.98E-03
rs4790508	A	C	CRK	3.37E-06	8.33E-03
rs308819	A	C	RAB38	4.00E-06	9.57E-03
rs308814	C	T	RAB38	4.00E-06	9.57E-03

### SOX2 regulates VDAC3 expression

Among the 16 SNP-gene pairs in SOX2 active patients that were not in SOX2 inactive patients, SNP rs798827 to gene VDAC3 pair caught our interest (Table 1). Voltage-dependent anion-selective channel 3 (VDAC3) are small integral membrane channels important for controlling the flux of metabolites between mitochondria and cytoplasm [33, 34]. It has previously been shown to play a role in apoptotic and oxidative stress signaling [35–37]. It was also found to be downregulated upon SOX2 silencing in two LSCC cell lines (Figure 3B).

**Figure 4:**
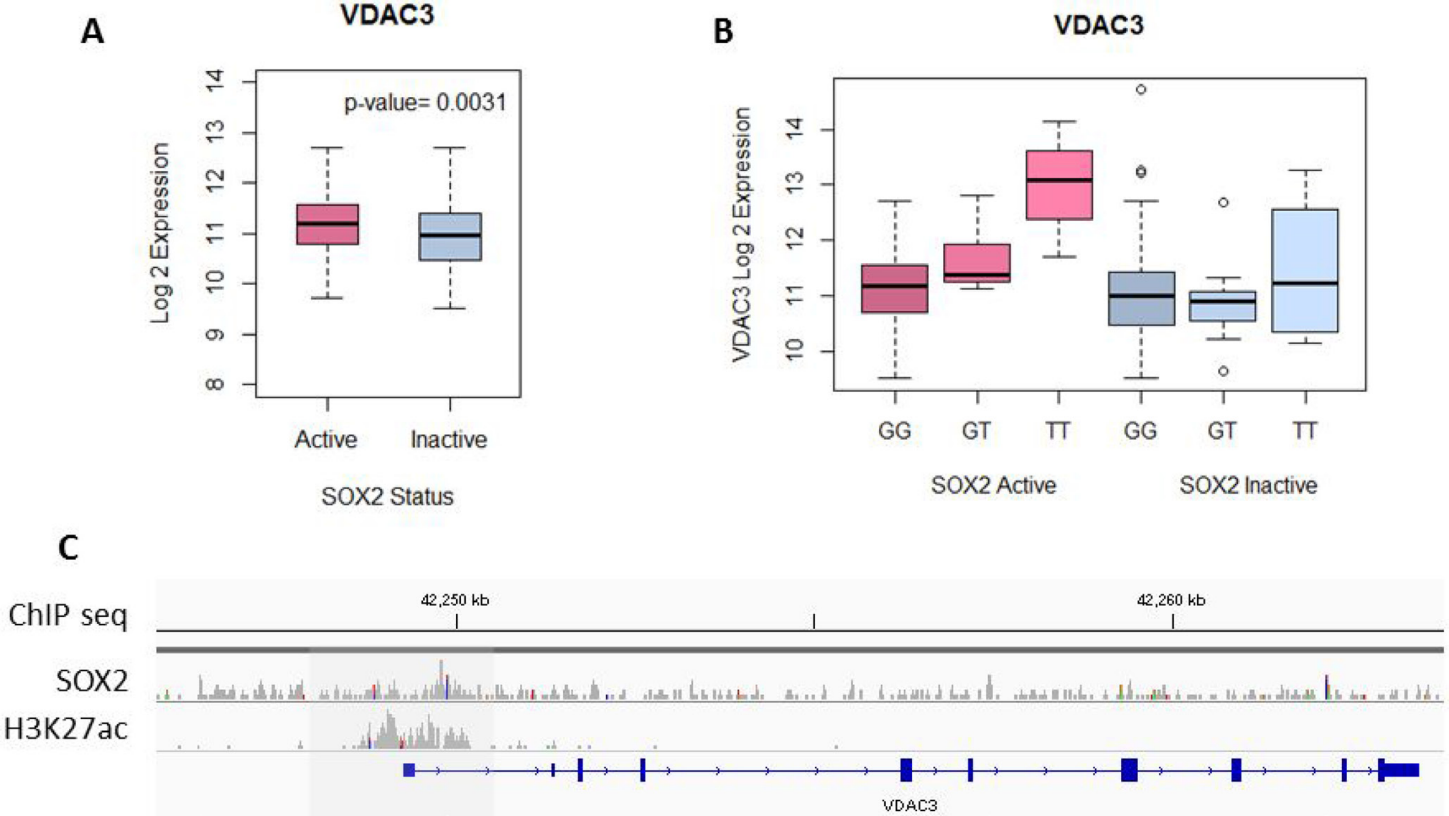
SOX2 regulates VDAC3 expression (**A**) VDAC3 expression is significantly higher in SOX2-active patients compared to SOX2-inactive patients, *t*-test *p* = 0.0031. (**B**) In SOX2-active patients, the SNP genotype is associated with a significant difference in VDAC3 expression, ANOVA *t*-test *p* = 6.91E-08. In SOX2-inactive patients, the difference is not present, ANOVA *t*-test *p* = 0.459. (**C**) SOX2 and H3K27ac ChIP-seq peaks for cell line HCC95 are shown for the promoter region of VDAC3.

Not only is the expression of VDAC3 significantly higher in SOX2 active patients when compared to SOX2 inactive patients (*p* = 0.0031), the expression of VDAC3 is also correlated with the genotype of SNP rs798827 in SOX2 active patients, but not in SOX2 inactive patients (ANOVA *t*-test *p* < 0.05) (Figure 4A and 4B). The T genotype of rs798827 correlated with a significantly higher expression of VDAC3 than the G genotype. This association is only seen in SOX2 active patients, but not in SOX2 inactive patients (Figure 4B). SOX2 ChIP-seq data from Watanabe H, *et al*. identified 5371 regions with high SOX2 interactions in a LSCC cell line (HCC95) [30]. SOX2 peaks were detected using MACS and normalized for copy number variation. Figure 4C shows the SOX2 ChIP-seq peak at the promoter region of VDAC3. ChIP-seq peaks for H3K27ac, an active enhancer mark, is also shown for the same LSCC cell line [38]. Collectively, our analysis indicates that SOX2 binds to the promoter region of VDAC3 and subsequently, regulates the expression of VDAC3.

### SNPs or LD SNPs are located in SOX2 binding motifs

**Figure 5:**
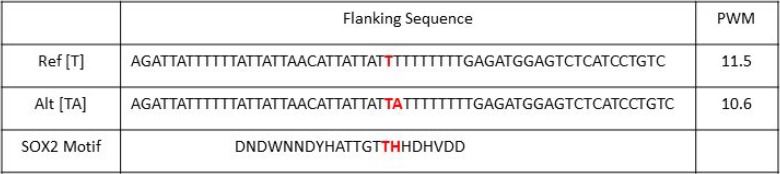
SNP rs58163073 alters the PWM of SOX2 The flanking sequences of rs58163073 from chromosome 8 position 42904940 +/– 29 nucleotides are shown for the reference and alternative alleles. The position weight matrix (PWM) score are obtained from HaploReg4.1. The reference [T] allele has a stronger binding affinity for SOX2 than the alternative [TA].

The HaploReg v4.1 web interface by Broad Institute is a well annotated database for SNP and their linkage disequilibrium (LD) SNPs [39–41]. When a SNP is in strong LD to another SNP, then those SNPs are highly associated with one another. In other words, the alleles of a few SNPs can suggest the alleles of their LD SNPs. Analyzing the 16 target SNPs from our cis-Matrix eQTL analysis of SOX2 active patients, we identified over 350 SNPs within strong LD (*r*^2^ > 0.80) to our 16 SNPs. SOX2 is a member of the SOX HMG box family of transcription factors [42]. It can bind to specific regions of the genome at consensus binding sequences. Looking at the flanking sequences of those LD SNPs, seven unique SNPs are located in and may alter a SOX family binding motif (Table 2). The SNP from rs798827-VDAC3 pair is within a strong LD to SNP rs58163073. This LD SNP is actually an insertion variation where an [A] nucleotide is inserted. The flanking sequences of LD SNP rs58163073 make up the SOX2 binding motif and the SNP genotype modified the position weight matrix score. The [T] allele has a stronger binding affinity for SOX2 (11.5) than the [TA] allele (10.6) (Figure 5). The [T] allele of rs58163073 is linked to the [T] allele of rs798827 which is correlated with a higher expression of VDAC3.

**Table 2:** Seven LD SNPs are located within and alters a SOX2 binding motif

SNP	LD SNP	Ref	Alt	D’	*r*^2^	AFR	AMR	ASN	EUR	Motifs
rs798827	rs58163073	T	TA	–1	1	0.7	0.83	0.85	0.97	Cart1, Dbx1, Foxa2, Foxp1, HDAC2, Ncx2, **Sox2**, Sox5, Zfp105, p300
rs4747471	rs199772546	TA	T	1	1	0.04	0.02	0.21	0	Arid3a2, Dbx1, Dbx2, FAC1, Foxa2, Foxa4, Foxj2, Foxk1, Foxo2, Foxp1, HNF1, Hlx1, Hoxa10, Hoxa5, Hoxc6, Hoxd8, Lhx3, Mef2, Msx-1, Nanog, Ncx2, Nkx6-1, PLZF, Pax-6, Pou2f2, Pou3f2, Pou3f4, Pou4f3, Prrx1, Sox13, Sox18, Sox19, **Sox2**, Sox5, Sox6, Sox7, Zfp105, p300
rs4747471	rs200774383	AAT	A	1	1	0.04	0.02	0.21	0	CDP7, Dbx1, Dbx2, Evi-1, FAC1, Foxa2, Foxa4, Foxj2, Foxk1, Foxo2, Foxp1, HNF1, Hlx1, Hoxa10, Hoxa5, Hoxd8, Lhx3, Lhx3, Mef2, Nanog, Ncx_2, Nkx6-1, Nkx6-2, PLZF, Pax-6, Pou2f2, Pou3f4, Pou6f1, Prrx1, Sox13, Sox18, Sox19, **Sox2**, Sox5, Sox6, Sox7, Zfp105, p300
rs4747471	rs7087230	C	T	1	1	0.3	0.06	0.28	0	Fox, Foxk1, Foxp1, Hoxa10, Hoxd8, Lhx3, Pou2f2, Sox18, **Sox2**, Sox3, Sox7, TATA
rs4747471	rs12217320	A	C	1	1	0.03	0.02	0.25	0	FAC1, Foxa4, Foxd3, Foxk1, Foxo1, Foxo2, Foxp1, HDAC2, Irf, Mef2, Nanog, RREB-1, Sox13, **Sox2**, Sox6, Sox7, Zfp105
rs4747471	rs1398027	G	C	1	1	0.002	0.02	0.25	0	**Sox2**
rs12057041	rs10968463	C	T	1	1	0	0.03	0.2	0.01	Foxj2, **Sox2**
rs10968456	rs10968463	C	T	1	1	0	0.03	0.2	0.01	Foxj2, **Sox2**

The eQTL SNP, LD SNP, and reference and alternative alleles of the LD SNPs are shown. The coefficient of linkage disequilibrium (D`) and the square of correlation coefficient (r^2^) are shown along with the frequency of the alternative allele in African (AFR), American (AMR), Asian (ASN), and European (EUR) populations. Motifs whose position weight matrix scores are affected by the LD SNPs are listed.

### VDAC3 and rs58163073 are located within the same TAD

**Figure 6:**
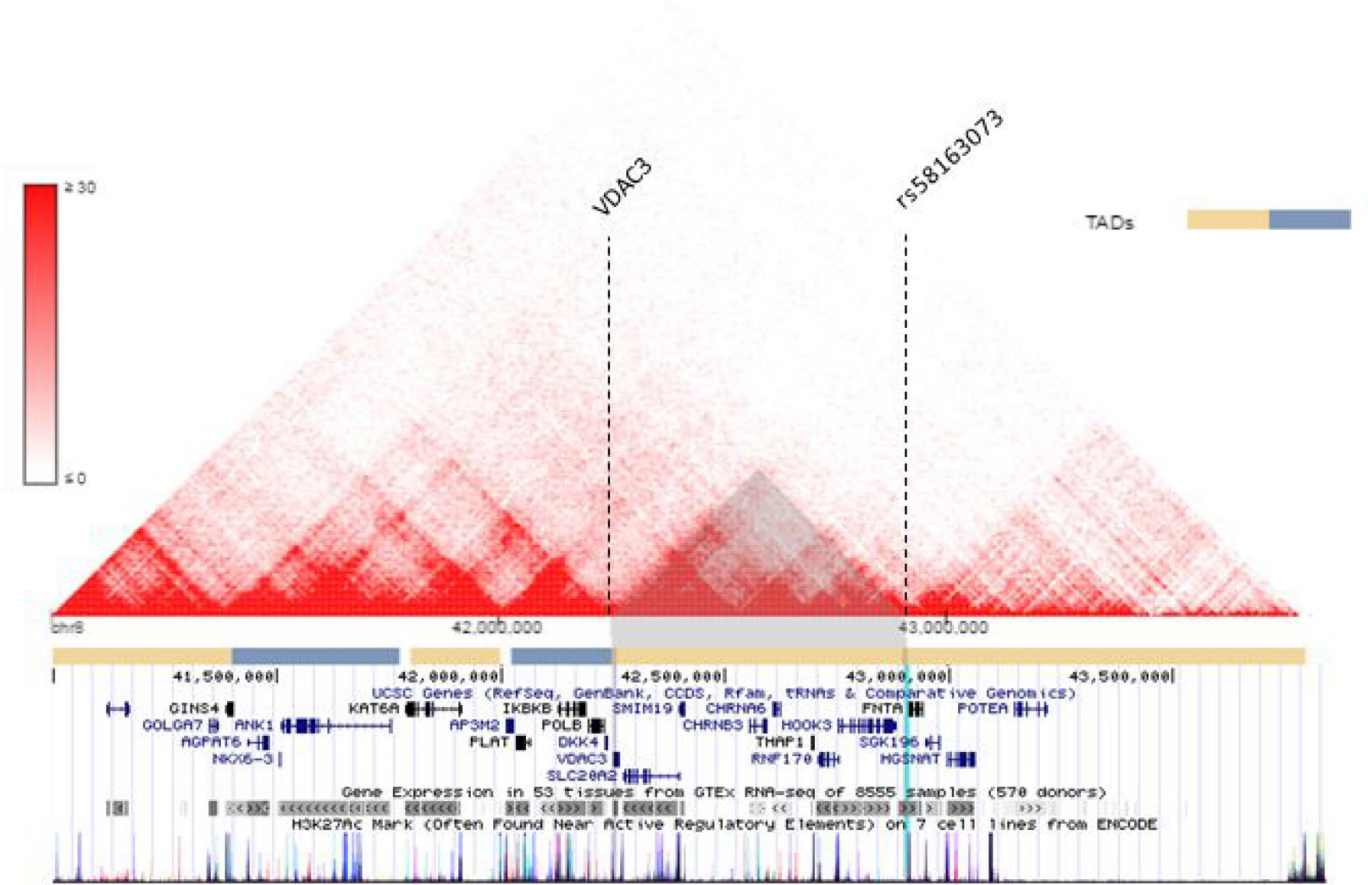
VDAC3 and rs58163073 are located within the same TAD in lung cell line The Hi-C heat map of lung cell line IMR90 is shown for chr8:41000000–44000000, resolution 10 kb. The location of VDAC3 and rs58163073 are indicated with dotted lines. The different TADs are marked with pale yellow and blue bars below the heat map. The locations of other genes are shown.

**Figure 7:**
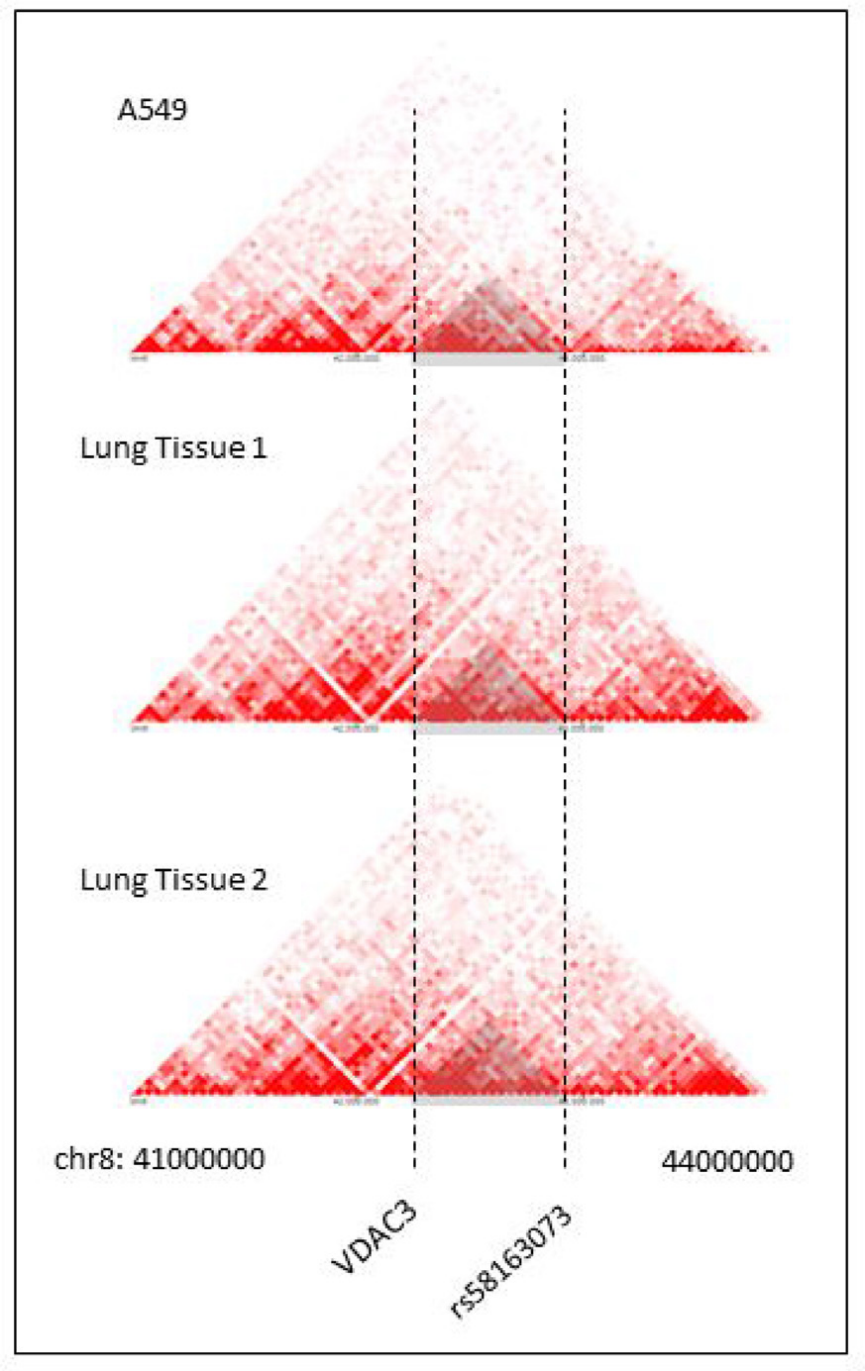
Hi-C heat maps for lung cancer cell line and lung tissues Hi-C heat maps of lung cancer cell line A549 and two lung tissue samples are show for chr8:41000000–44000000, resolution 40 kb. VDAC3 gene and SNP rs58163073 are marked with a dotted line.

The spatial organization of the genome plays a role in the transcriptional regulation of genes [43]. Chromatin conformation capturing methods such as Hi-C can reveal regions of the genome that have high interaction [44–46]. These regions are referred to as Topologically Associating Domains (TADs) [43, 47]. Hi-C data can be visualized in Hi-C heat maps [47]. Rao, S. S. P., Schmitt, A., and the Dekker Laboratory generated multiple Hi-C data for lung tissues and cell lines [29, 46, 48–50]. Figure 6 shows Hi-C heat map at chr8:41000000-44000000 for lung cell line IMR90. Figure 7 shows the Hi-C heap maps for lung cancer cell line A549, and two lung tissue samples. Figures were generated using 3D Genome Browser [51]. The location of VDAC3 and SNP rs58163073 are indicated. From analyzing Hi-C maps of lung cell lines (normal and cancer) and lung tissues, we have confirmed that VDAC3 and rs58163073 are located within the same TAD.

### Significant association between SNP and clinical features

**Figure 8:**
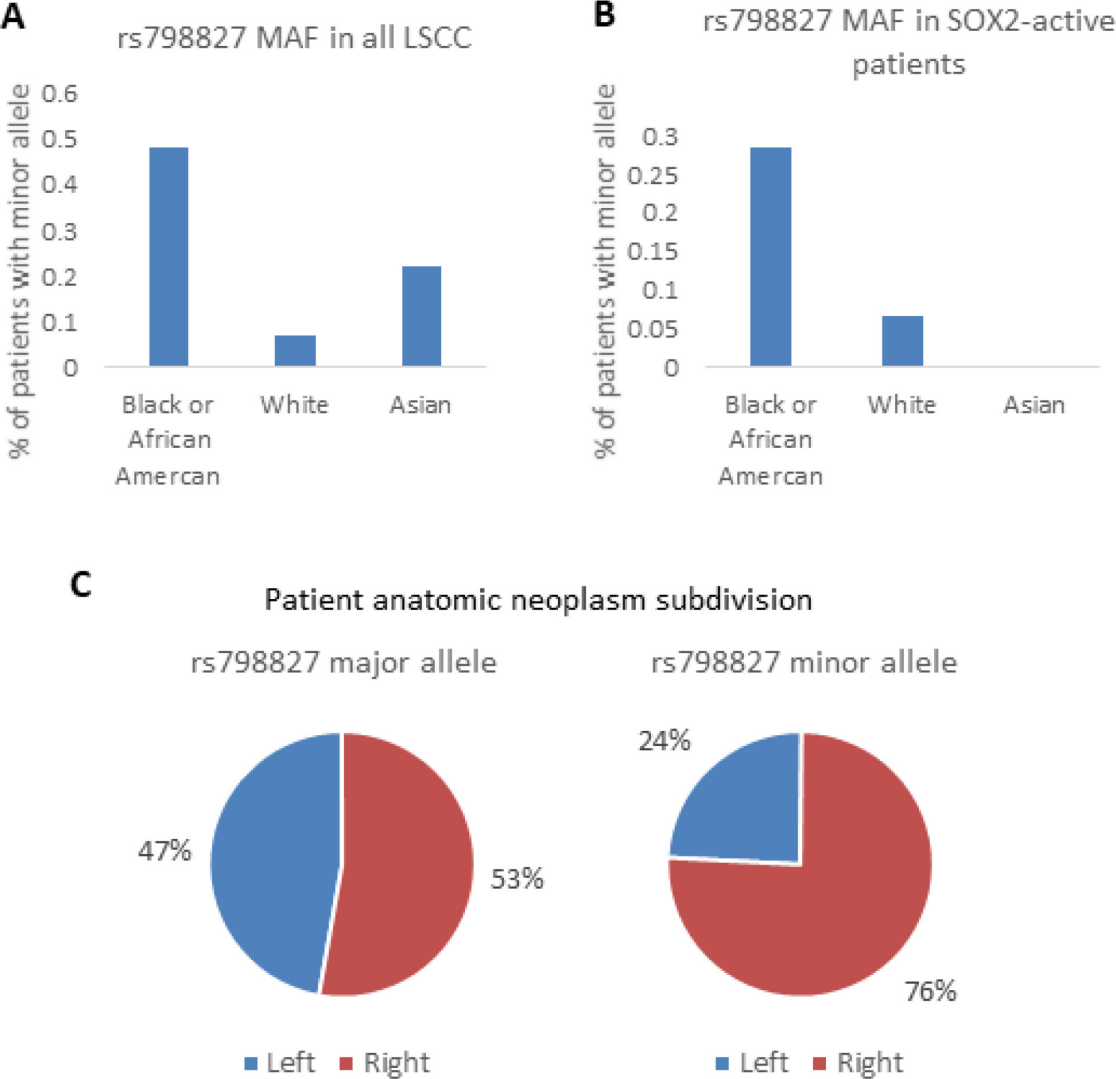
LSCC clinical analysis SNP rs798827 minor allele frequency (MAF) is shown for different races in LSCC patients (**A**) and in SOX2-active patients (**B**). Frequency of tumor location (left or right) is shown for patients with the major allele and minor allele (**C**).

Minor allele frequencies (MAF) of rs798827 and rs58163073 varied by race, with Black or African Americans more likely to carry the minor alleles than Whites and Asians. The MAF of rs798827 were 0.32 for Black or African American, 0.03 for White, and 0.15 for Asian according to information from the 1000 Genomes Project. These frequencies were found to be similar in LSCC patients, 0.48, 0.07, and 0.22 for each race respectively, again, with a higher frequency of the minor alleles in Black or African American patients (Figure 8A and 8B). Our SNP is also associated with location of tumor. The malignant location of patients with the major allele were 47% left and 53% right lungs. Patients with the minor allele had tumor more predominantly located on the right lungs (24 % left and 76% right, chi-square test 0.007461) Figure 8C. There were no significant association between SNP genotype, and stage and age at diagnosis (Table 3).

**Table 3:** Association of SNP rs798827 and clinical features in LSCC patients

	Major allele	Minor allele
**Race**		
**Black or African American**	5%	36%
**White**	93%	60%
**Asian**	2%	5%
**Location**		
**Left-Lower**	18%	14%
**Left-Upper**	30%	11%
**Right-Lower**	24%	32%
**Right-Upper**	29%	43%
**Age**		
**Min**	39	40
**1st Q**	62	63
**Median**	68	69
**Mean**	67.22	67.72
**3rd Q**	73	74.75
**Max**	90	84
**Stage**		
**i**	48%	57%
**ii**	33%	25%
**iii**	17%	16%
**iv**	1%	2%

## DISCUSSION

LSCC remains a complex disease with many molecular alterations. SOX2 is often seen amplified in LSCC patients and plays an important role in the progression and development of LSCC, however its mechanisms are not very well understood. Utilizing multiple layers of data from genomic to epigenomic, we are able to depict a potential mechanism of SOX2 in LSCC. First, LSCC patients were separated into two groups: SOX2-active and SOX2-inactive based on their SOX2 gene expression, copy number variation, and methylation. By first distinguishing patients based on their SOX2 activity, the mechanisms of SOX2 can be better elucidated. Using Matrix eQTL analysis, we linked variations in gene expression to SNP genotypes. Differences in SNP genotypes, especially within regulatory elements or binding motifs, can affect the binding affinity of transcription factors such as SOX2, and alter downstream transcription of target genes.

In our study, we focused on eQTLs that are found in SOX2-active patients but not in SOX2-inactive patients. We identified a SNP-gene pair (rs798827-VDAC3) that is unique in SOX2-active patients. The genotype of rs798827 is significantly correlated to the expression of VDAC3 in SOX2-active patients but not in SOX2-inactive patients. Since SOX2 is a transcription factor that binds to specific regions of the genome, variations in the binding sequences may alter the binding affinity of SOX2 to that region. Using HaploReg 4.1, we identified multiple SNPs within a strong LD to our eQTL SNPs. We found that rs798827 is within strong LD to SNP rs58163073 and motif analysis indicates that SNP rs58163073 is located within the binding motif of SOX2. The [T] allele of SNP rs58163073 increased the position weight matrix score for SOX2 and the [TA] allele decreased it. This SNP directly alter the binding sequence of SOX2 and the [T] allele increased its binding affinity.

Our cell line and ChIP-seq analysis further suggests that SOX2 regulates VDAC3. When SOX2 was silenced in two LSCC cell lines, the expression of VDAC3 was also downregulated. In addition, SOX2 ChIP-seq peaks show SOX2 binding in the promoter region of VDAC3. SNP rs58163073 and VDAC3 are also located within the same TAD in lung tissues, normal lung, and lung cancer cell lines. VDAC3 is the least investigated isoform of voltage-dependent anion channels. They are localized in the mitochondrial outer members and are pore-forming structures that control the exchange of metabolites between the mitochondria and cytoplasm [33]. They also play crucial roles in oxidative stress, maintaining redox status, and mediating cytochrome c apoptosis [34, 52]. Other studies have reported that VDAC3-deficient cancer cells have reduced permeability for ADP/ATP and decreased mitochondrial membrane potential [53–55]. The pathways VDAC3 were most involved in were cancer and reproductive system disease, according to Ingenuity Pathway Analysis (IPA) [56]. Deletion of VDAC3 significantly increases cell resistance to anti-tumor agent Erastin, which targets VDAC2 and VDAC3 [57, 58].

**Figure 9:**
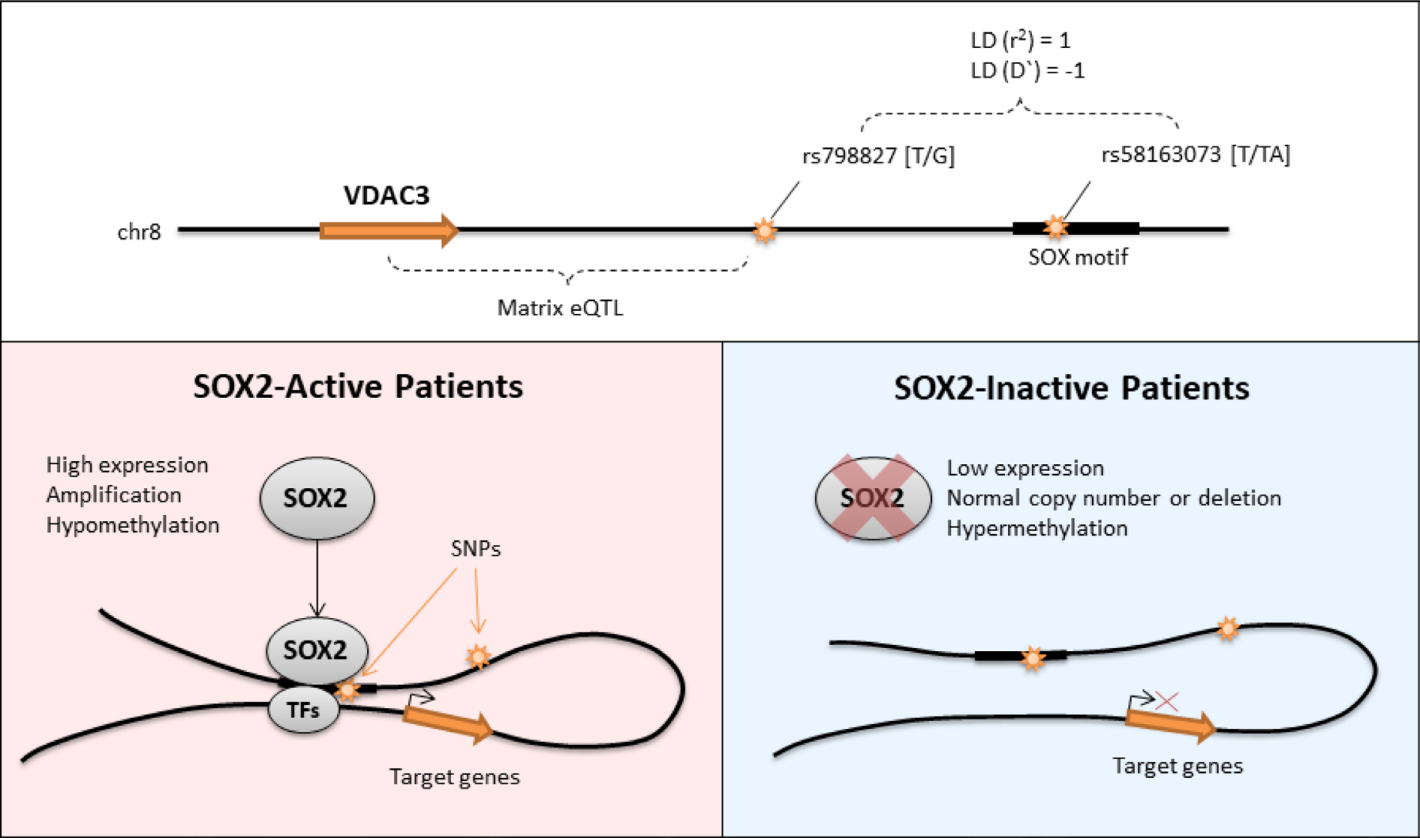
Graphical summary of the regulation of VDAC3 by SOX2 In SOX2-active patients, the genotype of SNP rs798827 is associated to the expression of VDAC3. This SNP is within strong LD to SNP rs58163073 which overlaps with the binding motif of SOX2. This mechanism is only seen in patients with SOX2 activity. Positions of genes and SNPs are not drawn to scale.

Further analysis shows that Black or African American patients have a higher MAF than those of White or Asian patients and patients with MAF had tumors located more predominantly on the right lungs. Collectively, these analyses support the notion that the SNP variant rs58163073 affects the binding of SOX2, which in turn affects SOX2's modulation of target genes such as VDAC3 (Figure 9). This discovery is found only when SOX2 activity is first considered. Our analysis can be used to understand the downstream mechanisms of transcription factors in other diseases and cancers.

## METHODS

### LSCC patient data

Patient gene expression, copy number variation, methylation, and SNP data were collected from The Cancer Genome Atlas (TCGA) database. Specifically, Illumina HiSeq RNAseqv2 was used for gene expression data, Genome Wide SNP 6.0 was used for both copy number variation and SNP genotype data, and Human Methylation 450 bioassay data set was used for methylation data. A total number of 366 LSCC patients had all three datatypes available.

### Matrix eQTL computation

Expression quantitative trait loci (eQTL) analysis was conducted with Matrix eQTL: Ultra-fast eQTL analysis via large matrix operations by Andrey Shabalin [26]. Software and resources were obtained from http://www.bios.unc.edu/research/genomic_software/Matrix_eQTL/. Output threshold p-values were set at 0.05. Local eQTLs (or cis eQTLs) were set at the default distance of 1 million base pairs of each other.

### SOX2 siRNA lung cancer cell line

SOX2 silencing experimental data was obtained from NCBI GSE48871 [32]. Two LSCC cell lines (H520 and LK2) were treated with either pooled siRNA sequences of SOX2 or scrambled control siRNAs) and their gene expression was profiled using Illumina's BeadChip Human HT-12v3 array. The gene expression data distribution was normalized to 0.

### Linkage disequilibrium SNPs and binding motifs

LD SNPs were explored using HaploRegv4.1 hosted by Broad Institute [39]. The database table is curated from information from the 1000 Genomes Project. Pairwise LD was calculated for all pairs of SNPs within 250 kb. A LD threshold of *r*^2^ > 0.8 was used to query variants. The 16 eQTLs were within strong LD to 263 SNPs. HaploRegv4.1 also contained information on regulatory motif changes. Only the SNP variants that overlapped the binding motif of SOX2 and changed the position weight matrix (PWM) score were considered. Under these conditions, only seven unique LD SNPs are identified.

### SOX2 and H3K27ac ChIP-seq in HCC95 cell line

SOX2 ChIP-seq data was obtained from NCBI- GSE46837 [30]. A LSCC cell line with SOX2 amplification was used in this study. SOX2 binding sites were detected using Model-based Analysis of ChIP-Seq (MACS) and normalized for copy number variation. There were 5371 peaks with high SOX2 interaction. H3K27ac ChIP-seq data was obtained from NCBI-GSE66992 [38]. LSCC cell line, HCC95 was also used and H3K27ac binding sites were called by MACs.

### Lung tissue and cell line Hi-C data

Hi-C data was obtained and visualized on 3D Genome Browser [59]. IMR90 Hi-C data was from Rao S, *et al.* (2014) [48], A549 cell line Hi-C data was from ENCODE Encyclopedia version 3 (2010) [29, 60], and two normal lung tissue Hi-C data were from Shmitt A, *et al*. (2016) [49]. Hi-C heat maps resolutions are at 10 kb, 40 kb, 40 kb, and 40 kb respectively, and between 41000000 and 44000000 position on chromosome 8, which is where VDAC3 gene and associated SNPs are located.

## SUPPLEMENTARY MATERIALS FIGURES AND TABLES


