# Supplementary material for: LSCC SNP variant regulates SOX2 modulation of VDAC3
Source: Oncotarget. 2018 Apr 27;9(32):22340–52. doi: 10.18632/oncotarget.24918 (PMC5976468; doi:10.18632/oncotarget.24918)
Supplement: Supplementary file 1 [file oncotarget-09-22340-s001.pdf]

LSCC SNP variant regulates SOX2 modulation of VDAC3

SUPPLEMENTARY MATERIALS

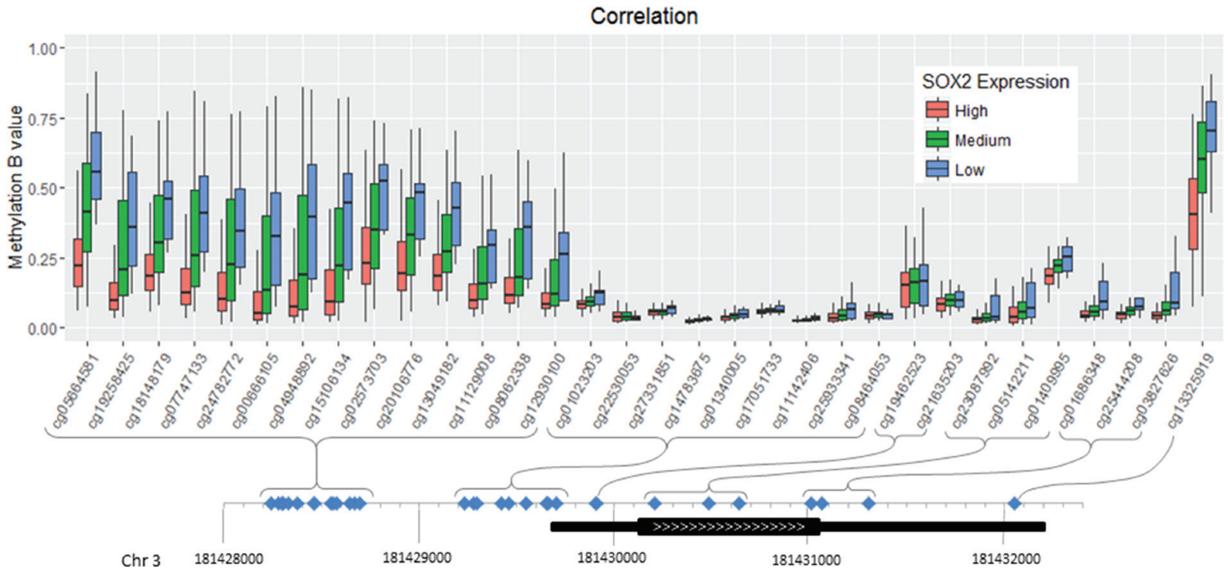

Supplementary Figure 1: Methylation β values for 32 SOX2-related CpG sites were examined in LSCC patients with high (n = 122), medium (n = 122), or low (n = 122) SOX2 gene expression. In all 32 sites, SOX2 expression was inversely correlated with methylation β values. The genomic location of the CpG sites is shown below. Only the first 14 CpG sites are considered in determining the overall methylation status of SOX2.

Supplementary Table 1: Model-based analysis of regulation of gene expression (MARGE) analysis of HCC95 H3K27ac ChIP-Seq data

Rank	Gene	Score	Rank	Gene	Score	Rank	Gene	Score	Rank	Gene	Score
1	CD44	189699.77	26	TM4SF1	50732.38	51	RN45S	46032.00	76	LTBR	35200.76
2	FJX1	189699.77	27	TNRC18	50679.97	52	KRT17	46032.00	77	CDKN1B	35156.14
3	EHF	189699.77	28	CAV1	50679.97	53	TXNRD1	45890.67	78	RPS21	34581.60
4	NFE2L2	189699.77	29	HES1	49662.93	54	AKR1B10	45890.67	79	C22orf26	34414.60
5	APIP	189699.77	30	SCNN1A	49442.34	55	MIR23A	45414.47	80	LOC150381	34252.45
6	PDHX	189699.77	31	LOC100128675	48753.18	56	SDC1	42671.31	81	LOC730091	34169.57
7	FLJ35776	189699.77	32	ALDH3A1	48182.53	57	PLEC	42357.95	82	TBL1XR1	33990.03
8	PTHLH	189699.77	33	KRT13	47224.15	58	IRF2BP2	39823.72	83	HIST1H2BF	33899.67
9	CAT	127763.12	34	FOXE1	46683.63	59	MIR27A	39156.91	84	NAT10	33824.86
10	PKP1	97244.88	35	CLIP4	46683.63	60	LOC727677	38740.68	85	HIST1H2AD	33520.88
11	MIR205HG	76837.13	36	DDR1	46683.63	61	MIR24-2	38200.49	86	HIST1H3D	33520.88
12	SLC20A2	76837.13	37	MALAT1	46683.63	62	BCL2L1	37779.46	87	MIR2117	33520.88
13	IER2	69792.78	38	LDLRAD3	46683.63	63	BCL9L	37417.72	88	IKBKB	33248.69
14	STX10	68970.01	39	SLC47A2	46683.63	64	LOC284454	37391.04	89	HIST1H4E	33248.69
15	TRIM44	68896.29	40	LAX1	46683.63	65	MIR661	37326.36	90	EGFR	33081.68
16	KRT42P	68896.29	41	FOSL2	46683.63	66	MIR4492	37080.82	91	AKR1C2	32320.20
17	FXYD3	68896.29	42	KRT19	46032.00	67	JUP	37009.58	92	PA2G4P4	31707.47
18	NRG1	66717.41	43	KRT5	46032.00	68	TNK2	36976.19	93	MYOF	31224.56
19	PAMR1	66717.41	44	TGIF1	46032.00	69	ST6GALNAC2	36962.04	94	DDX59	31224.56
20	MIR205	60390.76	45	DLG1	46032.00	70	LOC344887	36856.98	95	TLCD1	31162.34
21	RN5-8S1	53118.22	46	SOX2	46032.00	71	PHLDA3	35943.35	96	CAV2	31115.21
22	ABTB2	53118.22	47	MIR2278	46032.00	72	ZFHX3	35838.98	97	TNFRSF1A	31095.41
23	MIR3128	52973.59	48	ZMYND8	46032.00	73	KIF13A	35588.39	98	MIR4640	31075.60
24	LAMA5	51725.02	49	DLG1-AS1	46032.00	74	MIR21	35588.39	99	ZFP36	31075.60
25	KRT15	51725.02	50	GPR87	46032.00	75	PIM3	35483.63	100	IL1F10	31075.60

Regulatory potential was calculated using the MARGE-potential function as published by Wang, S, et. al. (2016) and the regulatory potential (RP) score is listed. Genes are ranks based on their RP scores, from highest to lowest. Only the top 100 unique genes are listed. SOX2 is bolded.

Supplementary Table 2: eQTLs (FDR *p*value < 0.01) in SOX2-inactive patients

SNP	AlleleA	AlleleB	Gene	*P* value	FDR
rs10505902	A	G	ETNK1	2.87E-17	3.30E-13
rs16925264	A	G	ETNK1	5.06E-14	4.28E-10
rs12299764	A	T	ETNK1	5.06E-14	4.28E-10
rs16925217	A	G	ETNK1	9.57E-13	7.31E-09
rs988175	C	G	ETNK1	5.13E-10	2.80E-06
rs16925483	A	G	ETNK1	5.13E-10	2.80E-06
rs12304738	A	G	ETNK1	5.13E-10	2.80E-06
rs7972877	C	T	ETNK1	5.13E-10	2.80E-06
rs7980969	A	C	ETNK1	5.13E-10	2.80E-06
rs6491171	C	T	CDK8	8.97E-10	4.69E-06
rs11046495	A	G	ETNK1	1.38E-09	6.98E-06
rs7134724	A	G	CPM	1.79E-09	8.84E-06
rs1047290	A	T	SKAP2	2.40E-09	1.17E-05
rs6956721	A	C	SKAP2	2.40E-09	1.17E-05
rs10505886	C	T	ETNK1	1.04E-08	4.35E-05
rs759931	A	G	ETNK1	1.46E-08	5.95E-05
rs12305233	A	C	ETNK1	1.46E-08	5.95E-05
rs2251988	C	T	ETNK1	9.33E-08	3.12E-04
rs16924949	A	G	ETNK1	1.02E-07	3.36E-04
rs4654947	C	T	NBPF3	3.43E-07	9.74E-04
rs12306101	A	G	ETNK1	6.29E-07	1.66E-03
rs11046794	G	T	ETNK1	7.25E-07	1.87E-03
rs12298399	C	T	ETNK1	7.53E-07	1.94E-03
rs10279895	C	T	SKAP2	9.24E-07	2.30E-03
rs4002871	A	T	CHL1	9.52E-07	2.36E-03
rs16891267	C	T	VDAC3	1.28E-06	3.04E-03
rs11046707	C	T	ETNK1	1.57E-06	3.62E-03
rs7309425	C	G	ETNK1	1.62E-06	3.70E-03
rs11990074	C	T	VDAC3	1.77E-06	4.00E-03
rs2699843	C	T	ETNK1	1.91E-06	4.26E-03
rs10478632	A	G	ZNF608	2.03E-06	4.47E-03
rs7975461	C	T	ETNK1	3.55E-06	7.10E-03
rs4325353	A	C	ETNK1	3.94E-06	7.71E-03
rs16925588	C	T	ETNK1	4.53E-06	8.66E-03
rs1363501	A	G	GALNT10	4.79E-06	9.09E-03
rs16925627	A	G	ETNK1	5.00E-06	9.42E-03
rs17082292	C	T	CDK8	5.15E-06	9.62E-03
rs4402409	C	T	CDK8	5.15E-06	9.62E-03
